# Elucidating the clinical spectrum and molecular basis of HYAL2 deficiency

**DOI:** 10.1016/j.gim.2021.10.014

**Published:** 2021-11-30

**Authors:** James Fasham, Siying Lin, Promita Ghosh, Francesca Clementina Radio, Emily G. Farrow, Isabelle Thiffault, Jennifer Kussman, Dihong Zhou, Rick Hemming, Kenneth Zahka, Barry A. Chioza, Lettie E. Rawlins, Olivia K. Wenger, Adam C. Gunning, Simone Pizzi, Roberta Onesimo, Giuseppe Zampino, Emily Barker, Natasha Osawa, Megan Christine Rodriguez, Teresa M. Neuhann, Elaine H. Zackai, Beth Keena, Jenina Capasso, Alex V. Levin, Elizabeth Bhoj, Dong Li, Hakon Hakonarson, Ingrid M. Wentzensen, Adam Jackson, Kate E. Chandler, Zeynep H. Coban-Akdemir, Jennifer E. Posey, Siddharth Banka, James R. Lupski, Sarah E. Sheppard, Marco Tartaglia, Barbara Triggs-Raine, Andrew H. Crosby, Emma L. Baple

**Affiliations:** 1 Medical Research, Research, Innovation, Learning and Development (RILD) Wellcome Wolfson Centre, College of Medicine and Health, University of Exeter Medical School, Royal Devon and Exeter NHS Foundation Trust, Exeter, United Kingdom; 2 Peninsula Clinical Genetics Service, Royal Devon and Exeter NHS Foundation Trust, Exeter, United Kingdom; 3 Department of Biochemistry and Medical Genetics, Rax Rady College of Medicine, Rady Faculty of Health Sciences, University of Manitoba, Winnipeg, Manitoba, Canada; 4 Genetics and Rare Diseases Research Division, Ospedale Pediatrico Bambino Gesù (Bambino Gesù Pediatric Hospital), IRCCS, Rome, Italy; 5 Genomic Medicine Center, Children’s Mercy Hospital, Kansas City, MO; 6 Pediatric Cardiology, Cleveland Clinic, Cleveland, OH; 7 New Leaf Center, Clinic for Special Children, Mount Eaton, OH; 8 Center for Rare Disease and Congenital Defects, Fondazione Policlinico Universitario A. Gemelli (Gemelli University Hospital), IRCCS, Rome, Italy; 9 MGZ Medical Genetics Centre, Munich, Germany; 10 Division of Human Genetics, Children’s Hospital of Philadelphia, Philadelphia, PA; 11 Golisano Children’s Hospital and Flaum Eye Institute, University of Rochester Medical Center, Rochester, NY; 12 Center for Applied Genomics, Children’s Hospital of Philadelphia, Philadelphia, PA; 13 GeneDx, Gaithersburg, MD; 14 Manchester Centre for Genomic Medicine, St Mary’s Hospital, Manchester University NHS Foundation Trust, Manchester, United Kingdom; 15 Division of Evolution, Infection and Genomics, School of Biological Sciences, Faculty of Biology, Medicine and Health, University of Manchester, Manchester, United Kingdom; 16 Department of Molecular and Human Genetics, Baylor College of Medicine, Houston, TX; 17 Human Genome Sequencing Center, Baylor College of Medicine, Houston, TX; 18 Department of Pediatrics, Baylor College of Medicine, Houston, TX; 19 Texas Children’s Hospital, Houston, TX

**Keywords:** Congenital heart disease, Facial dysmorphism, Hyaluronidase, Myopia, Orofacial clefting

## Abstract

**Purpose:**

We previously defined biallelic *HYAL2* variants causing a novel disorder in 2 families, involving orofacial clefting, facial dysmorphism, congenital heart disease, and ocular abnormalities, with *Hyal2* knockout mice displaying similar phenotypes. In this study, we better define the phenotype and pathologic disease mechanism.

**Methods:**

Clinical and genomic investigations were undertaken alongside molecular studies, including immunoblotting and immunofluorescence analyses of variant/wild-type human HYAL2 expressed in mouse fibroblasts, and in silico modeling of putative pathogenic variants.

**Results:**

Ten newly identified individuals with this condition were investigated, and they were associated with 9 novel pathogenic variants. Clinical studies defined genotype–phenotype correlations and confirmed a recognizable craniofacial phenotype in addition to myopia, cleft lip/palate, and congenital cardiac anomalies as the most consistent manifestations of the condition. In silico modeling of missense variants identified likely deleterious effects on protein folding. Consistent with this, functional studies indicated that these variants cause protein instability and a concomitant cell surface absence of HYAL2 protein.

**Conclusion:**

These studies confirm an association between *HYAL2* alterations and syndromic cleft lip/palate, provide experimental evidence for the pathogenicity of missense alleles, enable further insights into the pathomolecular basis of the disease, and delineate the core and variable clinical outcomes of the condition.

## Introduction

We first identified abnormalities in HYAL2 function through studies of a novel syndromic form of orofacial clefting in 5 Amish and 2 Saudi Arabian individuals^1^ associated with 2 distinct homozygous *HYAL2* missense variants. Affected individuals displayed a remarkably consistent pattern of craniofacial dysmorphism involving bilateral/unilateral cleft lip and palate, penetrant in all but 1 Saudi Arabian child, along with more variable features, including congenital cardiac anomalies (*cor triatriatum sinister*), myopia with staphyloma, cataract, conductive/sensorineural hearing loss, pectus excavatum, and single palmar creases. Consistent with the human condition, *Hyal2*^−/−^ mice displayed similar craniofacial abnormalities with submucosal cleft palate, and in a proportion of mice, *cor triatriatum sinister* and hearing loss were identified.^1^ To date, no further affected individuals have been described.

HYAL2 is a cell-surface protein proposed to act as a hyaluronidase enzyme in concert with HYAL1, the other major mammalian hyaluronidase acting in somatic tissues, catalyzing the degradation of the high molecular weight glycosaminoglycan polymer hyaluronan.^2,3^ Hyaluronan, a core constituent of the extracellular matrix, plays key roles in early development by providing the basement membrane for epithelial tissue formation.^4-6^ Hyaluronan is also a critical component of the developing heart and palatal shelf matrix,^7-9^ alongside important roles in other specialized tissues, including the vitreous humor of the eye^10^ and synovial fluid of synovial joints.^11^ *HYAL1* alterations are associated with mucopolysaccharidosis type IX (OMIM 601492), which is characterized by short stature, craniofacial dysmorphism, submucosal cleft palate, and joint abnormalities.^12^ The potential parity between HYAL2 and HYAL1 disruption supports the importance of maintained hyaluronan modeling for normal craniofacial and palatal shelf development. In this study, we describe the clinical, genetic, and molecular findings associated with putative *HYAL2* pathogenic variants in 10 individuals from 6 families of Amish, Romanian, Italian, and North European ancestry.

## Materials and Methods

### Patient ascertainment and genetic studies

Affected individuals were identified by their clinician and using GeneMatcher (Baylor-Hopkins Center for Mendelian Genomics - https://genematcher.org, accessed June 15, 2021). Phenotypic information was obtained by the clinical care provider using a targeted questionnaire with informed consent. DNA was extracted from blood/buccal samples using standard techniques for exome/genome sequencing. Variants with <5 reads, a frequency of >1% in the Genome Aggregation Database (gnomAD, Broad Institute) v2.1.1 and/or in-house databases were excluded. Exonic or intron/exon boundary (±6 nucleotides of the splice junction) de novo, homozygous, or compound heterozygous variants were evaluated and prioritized by call quality, allele frequency (minor allele frequency), inheritance pattern, and predicted functional consequence as previously described at Baylor College of Medicine (Individual 2; research trio exome sequencing),^13^ Ospedale Pediatrico Bambino Gesù (Individual 3; research trio exome sequencing),^14-16^ Children’s Mercy Kansas (Individual 4; research trio genome sequencing),^17^ GeneDx (Individual 6; diagnostic exome),^18^ Children’s Hospital of Philadelphia (Individual 7; research trio exome sequencing),^19^ and through the 100,000 Genomes Project (Individual 8; research trio genome sequencing, with gene-agnostic variant filtering) (see Supplemental text).^20^ Cosegregation studies were undertaken using dideoxy DNA sequencing (Supplemental Figures 1 and 2). *HYAL2* variants were deposited in ClinVar (SCV001572828-SCV001572838). For additional details about variant filtering methodologies, see Supplemental Data.

### Structural analyses

X-ray diffraction or nuclear magnetic resonance–derived structures of human HYAL2 (Q12891) were sought in UniProt and the Protein Data Bank to identify the 3-dimensional geometry and protein/substrate interactions of HYAL2 pathogenic missense variants, but no structures were found. Consequently, HYAL2 homologues were sought using the Basic Local Alignment Search Tool-Protein to identify a human HYAL1 (Q12794) structure (2PE4) with 43% sequence identity, including complete concordance in all HYAL2 putative pathogenic missense residues and >50% identity in a region surrounding the clustered missense variants (amino acids 130-280) (Supplemental Figure 3). A homology model (https://swissmodel.expasy.org/, accessed June 18, 2021) was visualized and annotated using Pymol 2.3 (Schrödinger LLC, 2019). Seven residues, previously shown to be critical for HYAL1 catalytic function, and identical in HYAL2, were annotated onto this model to highlight the probable enzymatic active site.^21^ We then modeled each HYAL2 amino acid substitution individually (FoldX5) to identify local predicted conformational changes that might perturb protein folding.^22^ Residues of interest were examined using PhosphoSitePlus (www.phosphosite.org, accessed June 15, 2021) to identify post-translational modifications.

The 8 putative pathogenic missense variants appeared clustered in 3-dimensional space (Supplemental Figure 4A) around the active site of HYAL1 (Supplemental Figure 4B). To quantify this, the mean (Euclidean) distance between amino acid substitutions was calculated using the spatial.-distance function (SciPy, Python 3). The mean Euclidean distance for this cluster of 8 variants was the mean of each pairwise comparison. A comparative distribution was derived by calculating the mean Euclidean distance of 8 randomly selected residues from the same model over 20,000 iterations (Supplemental Figure 4C).

### Plasmid recombinant constructs for variant functional studies

Putative pathogenic *HYAL2* variants (NM_003773.4: c.713T>G, c.611G>C, c.1271_1272delAC, c.194C>G, c.1273T>G, c.829C>T, c.883C>T) were introduced into the *HYAL2* complementary DNA expression vector pCMV6-XL5 by fusion polymerase chain reaction (PCR) to construct chimeric clones. Complementary primers specifying single-nucleotide variant substitutions or a deletion (Supplemental Table 1) were used in combination with outer primers (5′-atgcgggcaggcccaggccccacc-3′, forward; 5′-gctacaaggtccaggtaaaggcca-3, reverse) to generate *HYAL2* variants using Phusion High-Fidelity DNA Polymerase (New England BioLabs). PCR fragments were gel-purified and used as a PCR template using the outer primers for restriction digestion, and pCMV6-XL5 vector used for gel-purification, ligation, and transformation into electro-competent DH5α *E. coli*.^23^ Positive clones were cultured and plasmids were extracted and verified (dideoxy sequencing) at the Toronto Centre for Applied Genomics. A wild-type (WT) *HYAL2* vector with a 5′ deletion that does not express HYAL2 comprised the negative control.

### Human *HYAL2* expression analysis

*HYAL2* transient expression (4.0 μg plasmid) was performed in *Hyal2*^−/−^ mouse embryonic fibroblasts using TurboFect reagent (Thermo Fisher Scientific) in 6-well plates followed by immunoblot, immunofluorescence, or phosphoinositol-phospholipase C (PI-PLC) analysis. For immunoblotting, cells were collected 48 hours after transfection, washed with cold phosphate-buffered saline (PBS), and lysed (sonication). HYAL2 was detected by immunoblotting with rabbit anti-HYAL2 antibody (Proteintech AP-15115) and anti-rabbit horseradish peroxidase secondary antibody. Signals generated (Immobilon Western Chemiluminescence Kit, Millipore) were detected on a ChemiDoc imager (Bio-Rad Laboratories) with equal loading verification (β-actin).

For immunofluorescence, transfected cells were replated onto coverslips at 24 hours with HYAL2 detected in permeabilized and/or processed unpermeabilized cells (48 hours). Cells were washed (PBS), incubated (1% bovine serum albumin/PBS, 1 hour), and labeled (rabbit polyclonal anti-HYAL2 antibody, 1:250, 15115-1-AP; ProteinTech) for 2 hours at room temperature before detection (Alexa Fluor 568-conjugated donkey anti-rabbit secondary antibody, 1:500, A10042; Life Technologies) and Hoechst dye 33342 staining and imaging (Axio Imager.Z2, 63×/1.4NA oil objective).

To detect glycosylphosphatidylinositol (GPI)-anchored cell surface hHYAL2 at 48 hours after transfection, the entire population of cells in a transfected confluent well of a 6-well plate was washed (PBS ×2) and incubated (37 °C, 2 hours) with 0.5 U/mL PI-PLC (Sigma-Aldrich, P-5542) in serum-free Dulbecco’s Modified Eagle Medium supplemented with 25 mM 4-(2-hydroxyethyl)-1-piperazineethanesulfonic acid pH 7.4 (0.5 mL/well with gentle rocking). The medium from each well was collected and concentrated (Biomax 0.5 mL spin concentrators) for use as single samples. Sample proteins were separated (sodium dodecyl sulfate–polyacrylamide gel electrophoresis) and PI-PLC released cell surface HYAL2 was detected by immunoblotting.

## Results

### Clinical and genetic findings

**Figure 1:**
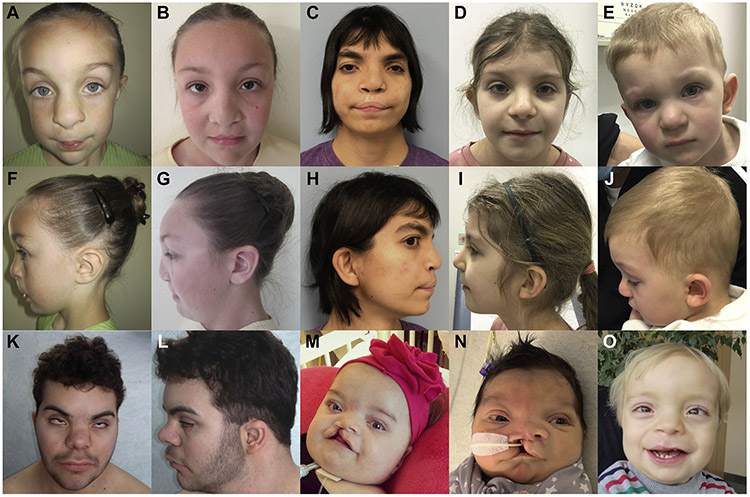
Facial features of individuals with HYAL2 deficiency, with new clinical photographs of individuals from Muggenthaler et al^1^ for comparison. Key features include frontal bossing, hypertelorism, a broad and flattened nasal tip, and cupped ears with overfolding of the superior helices. A, F. Individual XII:7 from Muggenthaler et al,^1^ (our Supplemental Figure 1 pedigree reference X:1). B, G. Individual XII:9 from Muggenthaler et al^1^ (our Supplemental Figure 1 pedigree reference X:3). C, H. Individual 6. D, I. Individual 8. E, J. Individual 10. K, L. Individual 3. M. Individual 4. N. Individual 5. O. Individual 7.

Table 1 summarizes the core phenotypic features of 10 individuals (2 Amish) aged 0 to 20 years with HYAL2 deficiency syndrome, alongside 7 individuals from Muggenthaler et al^1^ shown for comparison (Supplemental Table 2) (full clinical synopsis for each proband/family provided in Supplemental Data). Affected individuals share distinctive craniofacial similarities, including frontal bossing, hypertelorism, a broad and flattened nasal tip, and cupped ears with superior helices (Figure 1). Other common but variable clinical features included cleft lip and palate (both unilateral and bilateral); congenital cardiac anomalies, including valvular, atrial/ventricular septal defects; ocular features, including mild to severe myopia and cataracts; single palmar crease; and *pectus excavatum*.

In each newly reported affected individual, genomic studies (see Supplemental Data for description) identified pathogenic or likely pathogenic homozygous or compound heterozygous *HYAL2* variants as a likely cause of disease (for full American College of Medical Genetics and Genomics/Association of Molecular Pathology classifications refer to Supplemental Table 3). In Individuals 1 and 2, the pathogenic Amish *HYAL2* founder variant (Chr3 [GRCh38]:g.50320047T>C, NM_003773.4:c.443A>G; p.[Lys148Arg]), defined in our previous studies^1^ and with an allele frequency of 0.6% in the Anabaptist variant server comprising >10,000 exomes, was identified (Supplemental Figure 1). The homozygous Amish founder *MTPAP* variant (Chr10[GRCh38]:g.30313926T>C, NM_018109.3:c.1432 A>G; p.[Asn478Asp]), marked as pathogenic by ClinVar (VCV000018391.1) and known to cause mild-moderate developmental delay, optic atrophy, and spastic ataxia, was also identified in Individual 2, likely explaining these phenotypic features.^24^ Compound heterozygous novel pathogenic missense and nonsense *HYAL2* variants were identified in Individual 3 (Chr3[GRCh38]:g.50319661G>A, NM_003773.4:c.829C>T; p.[Arg277Cys], Chr3[GRCh38]:g.50319607G>A, NM_003773.4:c.883C>T; p.[Arg295*]). In siblings, Individuals 4 and 5, compound heterozygous pathogenic nonsense and missense *HYAL2* variants were identified (Chr3[GRCh38]:g.50320296G>C, NM_003773.4:c.194C>G; p.[Ser65*] Chr3[GRCh38]:g.503 18278A>C, NM_003773.4:c.1273T>G; p.[Phe425Val]). The nonsense variant is located in exon 2/4 with the resulting transcript expected to undergo nonsense-mediated decay. In Individual 6, a likely pathogenic homozygous *HYAL2* missense variant (Chr3[GRCh38]:g.50319879C>G; NM_003773.4:c.611G>C; p.[Gly204Ala]) was identified alongside a homozygous *RP2* missense variant (ChrX [GRCh38]:g.46837108G>C, NM_006915.2:c.8G>C; p.[Cys3Ser]), reported as likely pathogenic by ClinVar (accession; VCV000418458.2), potentially explaining the severity of this individual’s ocular phenotype.^25^ In Individual 7, compound heterozygous pathogenic frameshift and likely pathogenic missense *HYAL2* variants were identified (Chr3[GRCh38]:g.50318279_50318280delGT, NM_003773.4:c.1271_1272delAC; p.[His424Leufs*12], Chr3[GRCh38]:g.50319777A>C, NM_003773.4:c.713T>G; p.[Leu238Arg]). The frameshift alteration is predicted to cause a premature stop codon in the final exon (4/4) and escape nonsense-mediated decay, producing a truncated protein (436/473 amino acids). In Individuals 8, 9, and 10, two likely pathogenic missense *HYAL2* variants *in trans* were identified (Chr3[GRCh38]:g.50318419G>A, NM_003773.4:c.1132C>T; p.[Arg378Cys], and Chr3[GRCh38]:g.50320300C>T, NM_003773.4:c.190G>A; p.[Ala64Thr]).

All variants cosegregated as expected for an autosomal recessive disorder (Supplemental Figures 1 and 2) and were absent or rare in gnomAD (allele frequency <0.0001, Supplemental Table 4). All missense alterations affected evolutionarily conserved amino acid residues (Supplemental Figure 5) and were predicted deleterious (Supplemental Table 4).

### Expression of *HYAL2* variants

**Figure 2:**
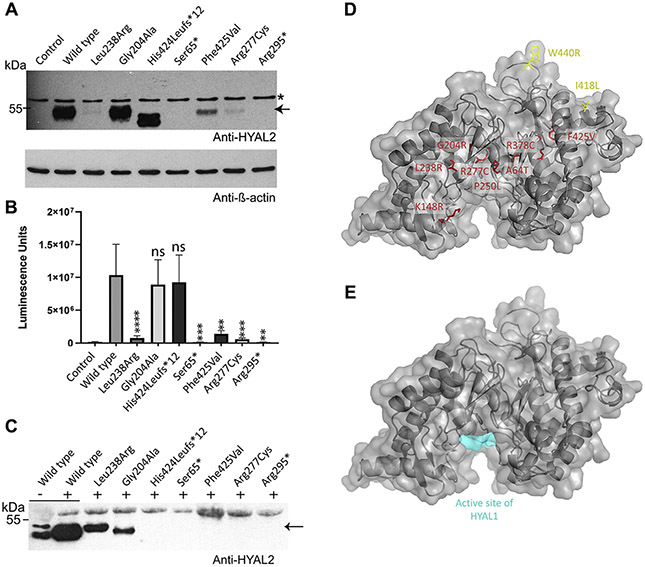
Functional studies and protein modeling of pathogenic HYAL2 variants reported to date. A. Expression of HYAL2 in *Hyal2*^−/−^ mouse embryonic fibroblasts (MEFs). Immunoblot analysis of protein lysates (10 μg per lane) prepared from transfections with HYAL2 expression vectors were analyzed with anti-HYAL2 (upper panel) and anti-β-actin (lower panel). HYAL2 is indicated by an arrow, and the asterisk indicates a cross reacting band that is evident even in the control. A second band, resulting from N-linked glycosylation of HYAL2, is apparent for His424Leufs*12. This band disappears after treatment with PNGase F (Supplemental Figure 6). The control was transfected with a vector that does not express HYAL2. B. Quantification of the immunoblots. The average ± SEM of the luminescence units from 4 separate transfection experiments are shown by the bars. The significance for each variant compared with the wild type (WT) was determined using a paired *t* test using the ratios from each experiment. *P* ≤ .0001 (****); *P* ≤ .001 (***); *P* ≤ .01 (**). C. Immunoblot analysis of HYAL2 released by phosphoinositol-phospholipase C (PI-PLC) treatment. *Hyal2*^−/−^ MEFs that were transfected with WT HYAL2 and HYAL2 variants were incubated with (+) or without (−) phospholipase C to release the cell surface HYAL2. HYAL2 was detected by immunoblot after the protein in the media was concentrated from the entire population of cells in a confluent well of a 6-well plate. This blot is representative of 3 independent experiments. Apparent difference in molecular weight between Gly204Ala and Leu238Arg result from differential N-linked glycosylation (Supplemental Figure 6B). A shorter exposure image (Supplemental Figure 6C) confirmed the highly abundant WT (+) band to be singular. No quantification was performed owing to the possibility of variability in PI-PLC efficiency significantly affecting these values. D. HYAL2 three-dimensional (3D) homology modeling using a crystal structure of HYAL1(2PE4) showing the position of described variants. Pathogenic variants are highlighted in red; variants present in a homozygous state in gnomAD v2.1.1 are colored yellow. E. The previously experimentally determined active site of HYAL1 shown on the same HYAL2 3D homology model as shown in D. ns, not significant.

*HYAL2* gene variants identified in Individuals 3 to 7 were introduced into human *HYAL2* complementary DNA to assess the impact on HYAL2 levels after transient expression in *Hyal2*^−/−^ mouse embryonic fibroblasts, with functional studies of p.(Lys148Arg) and p.(Pro250Leu) having been performed in previous work.^1^ Protein levels were compared with WT HYAL2 by transient transfection and immunoblot analysis. The Ser65* and Arg295* premature terminations resulted in no detectable HYAL2 protein, whereas the Leu238Arg, Phe425Val, and Arg277Cys alterations displayed very low protein levels, suggesting accelerated degradation (Figure 2A). Two variants, Gly204Ala and His424Leufs*12, were present at levels similar to WT protein (Figure 2B). The His424Leufs*12 variant resulted in 2 prominent forms of HYAL2 (Figure 2A), which when treated with PNGase F to remove N-linked glycans, resulted in a single protein band that was, as expected, smaller in size than the band from similarly treated WT HYAL2 (Supplemental Figure 6A).

### Impact of *HYAL2* variants on HYAL2 localization

**Figure 3:**
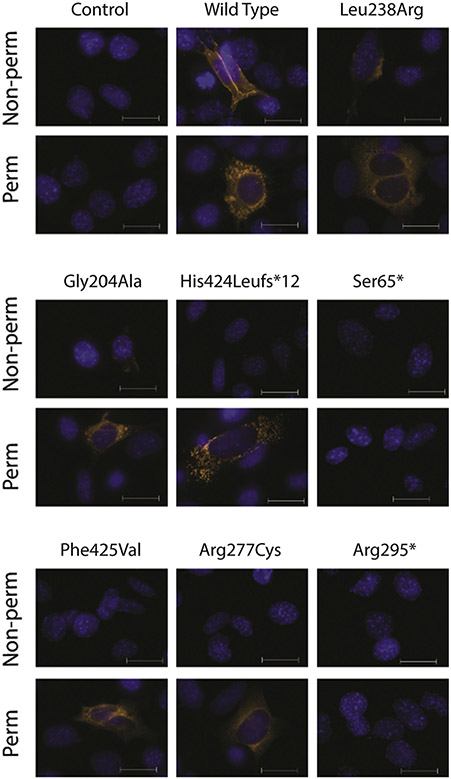
Comparative immunolocalization of *Hyal2*^−/−^ mouse embryonic fibroblasts (MEFs) expressing *HYAL2* variants. The transfected *Hyal2*^−/−^ MEFs were fixed and incubated with anti-HYAL2 primary antibody under non-perm and perm conditions. HYAL2 is detected with Alexa Fluor 568-conjugated donkey anti-rabbit secondary antibody and is labeled with orange fluorescence. Nuclei are stained blue with Hoechst. Scale bars, 20 μm. 63× magnification. Representative microscopy images from at least 3 independent experiments are shown. perm, permeabilized.

Proper HYAL2 processing includes modification by introduction of a GPI anchor for cell surface localization. Variants impacting protein folding may lead to endoplasmic reticulum (ER)-mediated degradation and/or a failure in C-terminal GPI anchor addition; such variants would be expected to reduce cell surface HYAL2 levels. To explore this hypothesis, we performed immunofluorescence studies using transfected cells under nonpermeabilized conditions (cell surface HYAL2) and permeabilized conditions (intracellular HYAL2) and analyzed HYAL2 released from the surface with phospholipase C (PLC) (Figure 2C). WT HYAL2 was abundantly detected at the cell surface and within cells using immunofluorescence (Figure 3). Importantly, no variant resulted in cell surface HYAL2 levels that were comparable with the levels in WT, although several variants enabled substantial expression of intracellular HYAL2. No intracellular or cell surface HYAL2 was detected for termination codons (p.[Ser65*] and p.[Arg295*]). Low levels of cell surface expression could be detected for p.(Leu238Arg) and p.(Gly204Ala).

### Removal of cell surface HYAL2 with PLC

To confirm the low levels of cell surface HYAL2 in cells expressing mutant *HYAL2* constructs, we released cell surface HYAL2 with PI-PLC (Figure 2C). Media collected from cells expressing WT HYAL2 were analyzed before and after treatment with PI-PLC. A low level of HYAL2 (−PLC) was detected in the medium, but this was much lower than the amount released after 2 hours of incubation with PI-PLC. The large number of cells used in this experiment allowed the clear confirmation of cell surface mutant HYAL2 that was only faintly apparent by immunofluorescence. Only 2 variants, Leu238Arg and Gly204Ala, led to substantial HYAL2 levels released from the cell surface, consistent with immunolocalization results. Interestingly, although WT HYAL2 has 2 forms that can be detected by immunoblot, 1 band predominated in the presence of each *HYAL2* variant. PNGaseF treatment of these samples showed that these 2 bands differ only in glycosylation (Supplemental Figure 6B), suggesting that the variants differently impact folding and glycosylation rates in the ER.

### *HYAL2* variant structural homology modeling

In total, 7 of 8 missense *HYAL2* variants affect residues located internally away from the HYAL2 molecular surface, making it unlikely that they directly bind ligands (Figure 2D). In all but 1 case (Ala64Thr), *in silico* analysis predicted these variants destabilize protein folding (Supplemental Table 5). In contrast, the only missense variants within our model that were present in homozygous state in gnomAD v2.1.1, Ile418Ser and Trp440Arg, were found at the protein periphery with side chains projecting outward and were either stabilizing or only very mildly destabilizing to protein folding. The Lys148Arg variant located on the protein surface is predicted to mildly encourage the folded state. However, because Lys148 entails the only known HYAL2 ubiquitinated residue,^26^ it may have a specific and key functional molecular role (eg, in signaling).

As expected, structural modeling defined a putative substrate binding cleft and the key catalytic residues in the same conformation as HYAL1 (Supplemental Figure 4A and B). Pathogenic HYAL2 residues were all identical in HYAL1 (Supplemental Figure 3), and notably, Lys148Arg is located within the predicted binding cleft. In total, 7 of 8 pathogenic missense variants cluster in the region of the substrate binding cleft (Supplemental Figure 4A and B), which is further supported by analysis of variant mean Euclidean distance in the lowest 3% (Z <−1.89) of all such groupings of this size in this protein (Supplemental Figure 4C).

## Discussion

The clinical and genetic studies described herein establish *HYAL2* gene variants as a cause of syndromic cleft lip and/or palate, and our molecular findings identify HYAL2 protein deficiency as the pathomolecular basis of disease. The comprehensive comparison with our previously reported cases, initially elucidated as founder alleles, define the core and variable clinical features that characterize the condition.

A recognizable pattern of craniofacial dysmorphism is invariably present, although a cleft lip and palate—previously considered to be a core clinical feature—is now shown through our larger cohort to be a common but more variable feature than previously thought. Auricular anomalies (cupped ears, overfolded helices), previously identified in a minority of individuals, are now noted in more than half of individuals and frontal bossing, a broad nasal tip, micrognathia, and *pectus excavatum* (Supplemental Figure 7) are considered variable features. Congenital cardiac anomalies, identified in most new cases, comprise an important component of the disease phenotype, and we identified a number of clinically significant anomalies likely to require surgical intervention in the first year of life (coarctation of the aorta, mitral valve atresia, hypoplastic left ventricle, pulmonary valve atresia, and tetralogy of Fallot). The presence of these severe phenotypes and the high overall incidence of cardiac defects in the affected individuals underscore the importance of cardiac screening at birth with pulse oximetry and echocardiography, allowing early detection of defects that may require surgical intervention to reduce the risk of morbidity and mortality associated with a delayed diagnosis^27^ (Supplemental Table 6).

Myopia, originally described in only 2 individuals in our initial publication, is now confirmed as another core clinical feature, being present in all individuals examined. This may be particularly severe (up to −16.75 dioptres) and associated with complications including myopic macular degeneration (4 individuals) and retinal detachment (2 individuals). Individual 6 displayed a particularly severe phenotype with high myopia leading to unilateral retinal detachment, potentially caused by coinheritance of a homozygous *RP2* variant previously described as pathogenic in a single hemizygous male with retinitis pigmentosa (OMIM 312600)^25^ but also present in 3 hemizygous males in gnomAD v.2.1.1. Additional ocular findings in some individuals, including posterior subcapsular cataract and wedge-shaped cortical cataract, suggest high myopia may be part of a hereditary vitreoretinal degeneration phenotype. The human vitreous is a specialized extracellular matrix composed of hyaluronan, the major macromolecule, interwoven with chondroitin sulfate proteoglycans (including versican and collagen IX) and other collagen fibrils.^10^ Versican binds to hyaluronan via a hyaluronan-binding domain located in the globular N-terminal region.^28^ Of note, pathogenic variants in *VCAN*, encoding versican, are associated with vitreoretinopathy, Wagner syndrome (OMIM 143200), and are implicated in congenital cardiac defects.^29^ These observations define HYAL2 as being critically important for ocular and cardiac development, with decreased hyaluronan and/or chondroitin sulfate degradation likely underlying ocular and cardiac disease in patients. In view of the association with high myopia and associated complications and the possible association with a more severe vitreoretinal degeneration phenotype, we suggest regular ophthalmic examinations from infancy to enable early detection and treatment of ocular pathologies (see Supplemental Table 6 for proposed clinical guidelines).

Hearing loss, noted in 5 of 7 of the originally described individuals, was present in only 2 of 9 new cases, possibly reflecting the reduced incidence of cleft palate, which is commonly associated with conductive hearing loss. However, screening for hearing loss in all patients at diagnosis is recommended because we cannot exclude an independent association with HYAL2 deficiency (Supplemental Table 6). Two affected individuals (2 and 6) in our study were considered to have cognitive impairment, although for individual 2 an explanatory second diagnosis was identified (MTPAP-related spastic ataxia). Individual 6 is also microcephalic, and it remains unclear whether these aspects of her condition are caused by a comorbid disorder. Importantly, for most patients, cognitive ability is unaffected, information that will aid genetic counseling for this condition (Supplemental Table 6).

This study also identified the first 3 individuals with a loss-of-function *HYAL2* variant *in trans* with a missense *HYAL2* variant (Individuals 3, 4, and 5) who notably exhibited cardiac anomalies at the more severe end of the spectrum (coarctation of the aorta, mitral valve atresia), with 1 also having severe myopia. These findings may indicate an emerging genotype–phenotype relationship regarding protein functionality and cardiac/ocular phenotype severity, which may ultimately aid refinement of clinical screening and enable the interpretation of missense alterations beyond variant of unknown significance.

HYAL2 is a cell-surface protein that undergoes complex cotranslational modification before achieving its final topology as a mature GPI-anchored cell surface glycoprotein. The c-translational steps required to generate mature HYAL2 begin in the ER, where, like other glycoproteins, even apparently conservative amino acid substitutions may lead to ER-associated degradation and HYAL2 deficiency. Our functional assays determined that the nonsense variants Ser65* and Arg295* lead to no detectable intracellular or cell surface HYAL2 protein (Figure 2 and 3). Although these shorter peptides are likely unstable in this transient overexpression system, in the endogenous situation the transcripts would likely be subjected to nonsense-mediated messenger RNA decay, leading to no HYAL2 protein. Our studies also revealed that Phe425Val, Leu238Arg, and Arg277Cys substitutions also result in low total levels of HYAL2, indicating mutant protein instability (Figure 2). Interestingly, expression levels of Gly204Ala and His424Leufs*12 mutant proteins remain similar to WT levels (Figure 2). As the His424Leufs*12 entails a 2 base pair deletion within the final exon of *HYAL2*, this is not unexpected given such last exonic alterations may escape nonsense-mediated decay and generate truncated albeit stable mutant protein products.^30,31^ Immunofluorescence and PI-PLC studies show that although some variants permit intracellular expression of mutant HYAL2, all impact HYAL2 levels at the cell surface, with reduced (Gly204Ala and Leu238Arg) or absent (Phe425Val, Arg277Cys, and His424Leufs*12) levels of mature HYAL2 detected or released at the cell surface (Figure 2 and 3). Also of note, while Leu238Arg is depleted intracellularly, it displays notable expression at the cell surface, although far below WT levels and similar to Gly204Ala. Interestingly, the Leu238Arg mutant protein has increased N-linked glycosylation, suggesting it has prolonged ER retention, possibly leading to a transient localization to the cell surface en route to the lysosome.^32^ Taken together, these findings show that all putative pathogenic variants assessed to date affect HYAL2 stability and/or HYAL2 functional deficiency.

Our homology models support these functional studies, suggesting that 6 of the 8 identified missense variants impact buried residues, which would be strongly deleterious to folding and likely lead to removal of mutant protein (Supplemental Table 5). Of these, Gly204Ala had the smallest predicted deleterious effect, consistent with our findings of normal protein expression levels and reduced (but not abolished) cell surface expression. One pathogenic variant, Lys148Arg, which was previously shown to reduce protein expression,^1^ was not predicted by homology modeling to be deleterious to folding. This residue represents the only HYAL2 ubiquitination site,^26^ marking the protein for degradation and trafficking; this likely explains the apparent deleterious nature of this variant. Furthermore, the altered side chain of the Lys148Arg substitution projects directly into the putative binding cleft and leads to altered hydrogen bonding (Figure 2D). *In silico* modeling also identified a clustering of 7 of the 8 missense variants near to the putative binding cleft, whereas variants present in the homozygous state in gnomAD were clustered at the periphery with side chains projecting outward. The importance of this clustering remains unclear because the protein levels are low in affected individuals.

HYAL2 is broadly expressed both in mouse and human tissues, and the significant parity in mouse and human phenotypes associated with HYAL2 deficiency has permitted new insights into the mechanistic basis of developmental defects in HYAL2-related disease. Despite the controversy surrounding the precise role of HYAL2 in hyaluronan turnover,^33,34^ tissues from mice deficient in HYAL2 show substantial accumulation of hyaluronan, indicating that HYAL2 is clearly involved in hyaluronan removal from the extracellular matrix.^35^ Studies have also shown that a failure to remove hyaluronan leads to increased epithelial-to-mesenchymal transition (EMT) and decreased differentiation.^36^ We hypothesize that this mechanism underlies the cardiac anomalies observed in mice and humans with HYAL2 deficiency. During cardiac development, EMT within cardiac cushions establishes the primordium that develops into cardiac valves and the ventricular septum.^37^ Removal of high molecular mass hyaluronan from the provisional extracellular matrix is shown to be critical to heart development^38,39^ such that increased hyaluronan in the absence of HYAL2 would lead to excess EMT and decreased differentiation affecting cardiac development. In keeping with this, HYAL2-deficient mice show increased numbers of mesenchymal cells in the heart and expanded cardiac valves and accessory tissues,^38,39^ and congenital cardiac defects are a major clinical feature of HYAL2 deficiency in humans.

A similar mechanism may underlie the craniofacial anomalies in HYAL2 deficiency. Overall, the craniofacial appearance of individuals carrying pathogenic *HYAL2* variants resembles the phenotypic spectrum of frontonasal dysplasias, with overlapping features, including hypertelorism, nasal anomalies, and midline orofacial clefts. These features can arise from defects in midline craniofacial development, a process involving the fusion of 3 separate facial processes through a combination of programmed cell death and conversion of epithelial cells into mesenchyme via EMT.^40^ Craniofacial bones of *Hyal2*^−/−^ mice show a central ossification defect, suggesting decreased differentiation in *Hyal2*^−/−^ embryonic tissues.^1^ This supports increased hyaluronan accumulation, which leads to improperly regulated EMT, and decreased differentiation as a common mechanistic pathway underlying some of the congenital craniofacial and cardiac abnormalities seen in both humans and mice deficient in HYAL2.

Although *Hyal2*^−/−^ knockout mice are viable (albeit with significant preweaning lethality and reduced survival),^1^ the absence of clear human knockouts in affected individuals identified to date may indicate that HYAL2 plays a crucial role in human growth and development, and its complete absence may be incompatible with human life. Although the specific developmental cascades impaired by HYAL2 deficiency remain to be identified, it is clear that HYAL2 function is important for cardiac and craniofacial development. Together, our genetic, functional, and structural modeling studies expand the molecular spectrum associated with pathogenic *HYAL2* variants and provide new insight into the likely disease mechanism and functional roles of HYAL2. Our comprehensive phenotyping assessments enable a clearer delineation of the core and variable phenotypical features of HYAL2 deficiency to be characterized. On the basis of the features described earlier, we have proposed a set of general guidelines to aid the clinical workup of patients affected by HYAL2 deficiency (Supplemental Table 6). The precise functions of HYAL2 in human growth remain incompletely understood, and further studies are important to elucidate the precise molecular and developmental roles of this molecule in cardiac, ocular, and craniofacial development.

## Supplementary Material

Supplemental

## Figures and Tables

**Table 1:** Clinical features of affected individuals with HYAL2 deficiency

Individual	1	2	3	4	5	6	7	8	9	10	Summary^c^
Genotype	p.(Lys148Arg) ∣ p.(Lys148Arg)	p.(Lys148Arg) ∣ p.(Lys148Arg)	p.(Arg277Cys) ∣ p.(Arg295*)	p.(Ser65*) ∣ p.(Phe425Val)	p.(Ser65*) ∣ p.(Phe425Val)	p.(Gly204Ala)∣p.(Gly204Ala)	p.(His424Leufs*12) ∣ p.(Leu238Arg)	p.(Arg378Cys)∣p.(Ala64Thr)	p.(Arg378Cys)∣p.(Ala64Thr)	p.(Arg378Cys)∣p.(Ala64Thr)	-
Ethnicity, Sex, Age^a^	Amish, F, 13.8 y	Amish, M, 9.4 y	Italian, M, 19 y	North European, F, died 10 mo	North European, M, died 10 d	Turkish, F, 20y	German, M, 4 y	Polish, F, 8.6 y	Polish, M, ToP	Polish, M, 4.3 y	-
Height, cm (SDS)^b^	152.5 (−0.93)	124.5 (−1.83)	163.5 (−1.75)	53 (+1.42)	51 (+0.23)	151 (−2.09)	NK	129.9 (−0.14)	NK	93.7 (1.12)	(Median: −0.93)
OFC, cm (SDS)^b^	55.5 (+0.51)	54.2 (−0.07)	53.7 (+0.51)	33.5 (−1.32)	35.5 (+0.23)	51 (−3.26)	NK	50.7 (−0.83)	NK	47 (−1.35)	(Median: −1.32)
Broad nasal bridge	✓	✓	✓	✓	✓	✓	✓	✓	NK	✓	13/14
Hypertelorism	✓	✓	✓	✓	✓	✓	✓	✓	NK	✓	13/16
External ear abnormalities	✓ Small, overfolded thickened helices	✓ Small, overfolded thickened helices	✓ Small, overfolded thickened helices	✓ Small, low set, small lobule, R ear pit	✓ Low set, cupped, thick helix, prominent tragus	✓ Small ear lobes, chronic otitis media	✓ Small, overfolded, thick helix, low set, small lobule	×	NK	✓ L preauricular pit	11/14
Cleft lip/palate	×	×	×	✓ R CLP	✓ R CLP	✓ Bilat. CLP	✓ R CLP	×	Not reported	×	10/17
Micrognathia	✓	✓	×	✓	✓	×	×	×	NK	×	9/14
Frontal bossing	✓	✓	×	×	✓	×	×	×	NK	×	5/14
Ptosis	×	×	✓	×	✓	×	×	×	NK	×	5/13
Cardiac anomalies	✓ Mild AS and AR	×	✓ CoA and VSD	✓ VSD, ASD, PDA, PHTN	✓ Complex	×	✓ ToF	✓ ToF	✓ Hypoplastic left heart	✓ DORV, VSD	12/17
*Pectus excavatum*	×	✓	✓	×	×	✓	×	×	NK	×	7/16
Single palmar crease	✓	✓	×	×	✓	×	✓	×	NK	✓	9/13
Myopia (refraction)	✓ Mild	NK	✓ Severe	✓ Mild	NK	✓ High	✓ High	✓ High	NK	Suspected	11/11
Cataract	×	×	✓ Bilat.	NK	NK	×	×	×	NK	×	2/8
Other ocular features		Poor vision	Myopic maculopathy, RD	PPM R VH		L RD with enucleation R macular atropy	Myopic maculopathy, rod dystrophy suspected		NK		-
Hearing loss	×	×	×	×	✓ Failed NBHS on L side	✓ R: mild-sev. SNHL L: mild-profound mixed	×	×	NK	×	7/16
Duodenal web	NK	NK	NK	✓	NK	NK	NK	×	NK	×	2
Other clinical findings	Broad halluces, broad and distally placed thumbs	Ataxia, mild/moderate developmental delay, broad halluces broad thumbs	Accessory oral frenulum low posterior hairline Short, webbed neck	Broad deviated thumbs, hypoplastic nails, prominent creases, bilat. extrarenal pelvises, congenital diaphragmatic hernia, glabellar capillary nevus, cystic hygroma	Cystic hygroma at 11 wk, hydrops, hypoplastic nails, small penis, bilateral undescended testicles, large anterior fontanelle	Intellectual disability, No speech until 3 y, autism spectrum disorder, ADHD, fingertip whorls, finger webbing, webbed neck	Broad halluces Cryptorchidism Short neck fifth finger clinodactyly	Broad thumbs	NK	Bilateral 2-3 toe syndactyly	-
Comorbid diagnosis		Spastic ataxia 4, autosomal recessive				Retinitis pigmentosa 2					

(✓) and (×) indicate presence and absence of a feature in an affected subject, respectively.

*ADHD*, attention deficit hyperactivity disorder; *AR*, aortic regurgitation; *AS*, aortic stenosis; *ASD*, atrial septal defect; *AV*, aortic valve, *Bilat.*, bilateral; *BMI*, body mass index; *CoA*, coarctation of aorta; *CLP*, cleft lip and palate; *D*, dioptres; *DORV*, double outlet right ventricle; *F*, female; *L*, left; *M*, male; *NBHS*, newborn hearing screen; *NK*, not known; *OFC*, occipitofrontal circumference; *PDA*, patent ductus arteriosus; *PHTN*, pulmonary hypertension; *PPM*, persistent pupillary membrane; *R*, right; *RD*, retinal detachment; *SDS*, SD score; *sev.*, severe; *SNHL*, sensorineural hearing loss; *ToF*, tetralogy of Fallot; *ToP*, termination of pregnancy; *Unilat*, unilateral; *VH*, vitreous hemorrhage; *VSD*, ventricular septal defect.

a Refers to decimal age of examination.

b Height, weight, BMI, and OFC Z-scores were calculated using a Microsoft Excel add-in to access growth references based on the LMS (Lambda Mu Sigma) method using a reference European population (https://www.healthforallchildren.com/, accessed September 3, 2021).

c Including 7 individuals from Muggenthaler et al^1^ (Supplemental Table 2).

## Data Availability

The variants listed in this paper have been deposited in the ClinVar database (https://www.ncbi.nlm.nih.gov/clinvar/) with accessions SCV001572828 - SCV001572838.
